# Coordinated Actions of Glyoxalase and Antioxidant Defense Systems in Conferring Abiotic Stress Tolerance in Plants

**DOI:** 10.3390/ijms18010200

**Published:** 2017-01-20

**Authors:** Mirza Hasanuzzaman, Kamrun Nahar, Md. Shahadat Hossain, Jubayer Al Mahmud, Anisur Rahman, Masashi Inafuku, Hirosuke Oku, Masayuki Fujita

**Affiliations:** 1 Molecular Biotechnology Group, Center of Molecular Biosciences, Tropical Biosphere Research Center, University of the Ryukyus, 1 Senbaru, Nishihara, Okinawa 903-0213, Japan; mhzsauag@yahoo.com (M.H.); h098648@eve.u-ryukyu.ac.jp (M.I.); okuhiros@comb.u-ryukyu.ac.jp (H.O.); 2 Department of Agronomy, Faculty of Agriculture, Sher-e-Bangla Agricultural University, Dhaka 1207, Bangladesh; anisur68@yahoo.com; 3 Department of Agricultural Botany, Faculty of Agriculture, Sher-e-Bangla Agricultural University, Dhaka 1207, Bangladesh; knahar84@yahoo.com; 4 Laboratory of Plant Stress Responses, Faculty of Agriculture, Kagawa University, Miki-cho, Kita-gun, Kagawa 761-0795, Japan; shahadatsau24@gmail.com (M.S.H.); jamahmud_bd@yahoo.com (J.A.M.); 5 Department of Agroforestry and Environmental Science, Faculty of Agriculture, Sher-e-Bangla Agricultural University, Dhaka 1207, Bangladesh

**Keywords:** abiotic stress, antioxidant defense, glutathione, methylglyoxal, oxidative stress, reactive oxygen species

## Abstract

Being sessile organisms, plants are frequently exposed to various environmental stresses that cause several physiological disorders and even death. Oxidative stress is one of the common consequences of abiotic stress in plants, which is caused by excess generation of reactive oxygen species (ROS). Sometimes ROS production exceeds the capacity of antioxidant defense systems, which leads to oxidative stress. In line with ROS, plants also produce a high amount of methylglyoxal (MG), which is an α-oxoaldehyde compound, highly reactive, cytotoxic, and produced via different enzymatic and non-enzymatic reactions. This MG can impair cells or cell components and can even destroy DNA or cause mutation. Under stress conditions, MG concentration in plants can be increased 2- to 6-fold compared with normal conditions depending on the plant species. However, plants have a system developed to detoxify this MG consisting of two major enzymes: glyoxalase I (Gly I) and glyoxalase II (Gly II), and hence known as the glyoxalase system. Recently, a novel glyoxalase enzyme, named glyoxalase III (Gly III), has been detected in plants, providing a shorter pathway for MG detoxification, which is also a signpost in the research of abiotic stress tolerance. Glutathione (GSH) acts as a co-factor for this system. Therefore, this system not only detoxifies MG but also plays a role in maintaining GSH homeostasis and subsequent ROS detoxification. Upregulation of both Gly I and Gly II as well as their overexpression in plant species showed enhanced tolerance to various abiotic stresses including salinity, drought, metal toxicity, and extreme temperature. In the past few decades, a considerable amount of reports have indicated that both antioxidant defense and glyoxalase systems have strong interactions in conferring abiotic stress tolerance in plants through the detoxification of ROS and MG. In this review, we will focus on the mechanisms of these interactions and the coordinated action of these systems towards stress tolerance.

## 1. Introduction

Due to global climate change, the frequency and severity of abiotic stresses on plants have been increasing. These stresses include salinity, drought, flooding, high temperature (HT), low temperature (LT), UV-radiation, ozone, and metal toxicity; even their occurrences are often sudden or unpredicted, which causes substantial losses in plant productivity [1,2]. All the abiotic stresses cause physiological and metabolic disorders and adversely affect plant phenological and developmental processes; thus, worldwide abiotic stresses are liable for a yield reduction of more than 50% [3,4].

One of the major consequences of abiotic stress is oxidative stress [5,6]. Impaired stomatal conductance, disruption of the photosynthetic apparatus or of pigments, malfunctioning of the Calvin cycle and photosystem, inactivation of the enzymes of photosynthesis including RuBisCO, reductions in carboxylation reaction efficiency, electron transport chain (ETC) efficiency, regeneration of NADP^+^, and increased photorespiration are some of the major reasons for the overproduction of reactive oxygen species (ROS) under abiotic stress [4,7]. Methylglyoxal (MG) is a highly reactive α,β-dicarbonyl ketoaldehyde, generated as a by-product of several metabolic pathways such as glycolysis, and can be produced from photosynthesis intermediates (glyceraldehyde-3-phosphate) and dihydroxyacetone phosphate. Methylglyoxal production increases 2–6-fold in many plant species under abiotic stress [8]. Reactive oxygen species are highly reactive, and MG is a potent reactive cytotoxin capable of a complete disruption of cellular functions, including the peroxidation of lipids, the oxidation of protein, the oxidation of fatty acids, and the disruption of biomembrane structures and functions [9,10]. To scavenge excessively produced ROS, plants possess antioxidant defense system composed of an array of non-enzymatic (ascorbic acid (AsA), glutathione (GSH), phenolic compounds, alkaloids, non-protein amino acids, and α-tocopherols) and enzymatic components (superoxide dismutase (SOD), catalase (CAT), ascorbate peroxidase (APX), glutathione reductase (GR), monodehydroascorbate reductase (MDHAR), dehydroascorbate reductase (DHAR), glutathione peroxidase (GPX), and glutathione *S*-transferase (GST) [4,10]. On the other hand, MG is detoxified via the glyoxalase system composed of glyoxalase I (Gly I) and glyoxalase II (Gly II), which catalyze the detoxification of MG to d-lactate using reduced glutathione (GSH) as a cofactor [8]. This is a two-step reaction. The first step, which is catalyzed by Gly I, involves the reaction of MG with GSH, resulting in the formation of hemithioacetal that is then converted to *S*-d-lactoylglutathione (SLG). In the second step, which is catalyzed by Gly II, GSH is regenerated and d-lactate is formed by the hydrolysis of SLG. At the end of the reaction, GSH is recycled because the availability of GSH is an important factor for detoxifying MG via the glyoxalase system [8].

Enhanced antioxidant defense systems have been reported to improve plant abiotic stress tolerance in different studies. However, the role of the glyoxalase system in relation to abiotic stress tolerance has been studied in a very narrow range [11,12,13,14,15,16,17,18,19,20,21,22,23,24,25]. Methylglyoxal can catalyze the photoreduction of O_2_ to O_2_^•−^ at photosystem I and increases oxidative stress [26]. Plants’ antioxidant defense systems and glyoxalase systems both use GSH-dependent pathways to detoxify ROS and MG, respectively. Therefore, the possibility of an interaction between ROS and MG detoxification systems can be brought under consideration. Some reports have indicated signaling function of ROS and MG in plants [27,28,29]. The glyoxalase system has been studied widely in animal systems. Glyoxalase I and Gly II have been purified and characterized from a few plant species, but their role is yet to be explored. Moreover, a very recent report explored the presence of Gly III proteins in plants, which performs the function of direct conversion of MG to d-lactate. Identification of this shorter route for MG detoxification is a signpost in the field of glyoxalase system research [25]. Moreover, the biological implication of this pathway under stress has received recent attention. In the present review, we shed light on the coordinated role of antioxidant defense and glyoxalase systems in relation to plant abiotic stress tolerance.

## 2. Oxidative Stress: A Major Consequence in Plants under Abiotic Stress

**Figure 1:**
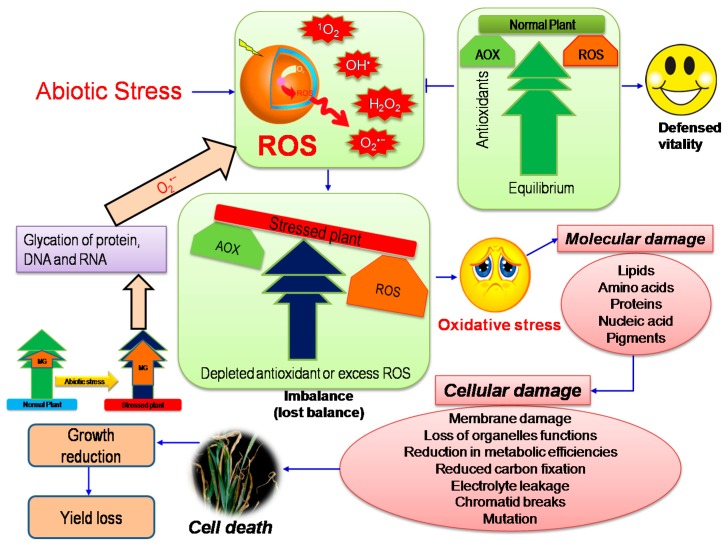
Generation of oxidative stress due to the consequences of abiotic stress (ROS, reactive oxygen species; ^1^O_2_, singlet oxygen; O_2_^•−^, superoxide anion; H_2_O_2_, hydrogen peroxide; OH^•^, hydroxyl radical; MG, methylglyoxal; AOX, antioxidants).

Abiotic stress is an unavoidable limiting factor for agriculture and is becoming a significant problem in the modern world. Plants grown under natural conditions are constantly subjected to a variety of abiotic stresses such as salinity, drought, toxic metal/metalloids, HT, LT, waterlogging/flooding, ozone, and UV radiation. [4]. Atmospheric oxygen as a product of photosynthesis has two great roles for aerobic organisms: firstly, it activates energy production; secondly, it forms ROS [30]. Reactive oxygen species are strong oxidizers and react with a large variety of biological molecules in plant cells. Reactive oxygen species are endlessly produced in plant cells as a result of aerobic metabolism in most intracellular organelles such as chloroplast, mitochondria, and peroxisomes [31]. In each aerobic cell, a dynamic equilibrium was observed between ROS generation and the antioxidant defense system ([4], Figure 1). Abiotic stress increases the ROS generation and disrupts the equilibrium in favor of oxidative reaction and creates oxidative stress [10]. In plant cells, chloroplast is the principal source of ROS generation. Under most stresses, in adequate energy indulgence is occurred in photosynthesis and a successive reduction of molecular oxygen yields ROS including singlet oxygen (^1^O_2_), superoxide anion (O_2_^•−^), hydrogen peroxide (H_2_O_2_), and hydroxyl radical (OH^•^) [31,32]. In addition, ROS have also overproduced in non-photosynthetic tissues such as roots, hypocotyls, or coleoptiles under stress, where O_2_^•−^ and H_2_O_2_ levels are increased in response to stress followed by a hyper sensitive reaction that leads to cell death. Hence, producing OH^•^ by cell wall–bound peroxidases is also related with other physiological responses such as the breakdown of cell wall structural polymers [31].

The mode of ROS production varies among stress types. Under salinity and drought stress, plants want to avoid excess water loss and reduce the stomatal conductance. As a result, internal CO_2_ concentration decreases, and the reduction of CO_2_ by the Calvin cycle becomes very slow. This means that salt or drought stress reduces the availability of CO_2_ and hinders carbon fixation. As a result, chloroplasts of cells become exposed to excessive excited energy and increase the production of different ROS [10]. This excess generation of ROS throughout salt or drought stress results from impaired electron transport procedures in the chloroplasts and mitochondria of plant cells. Reduction of the activity in photosystem II (PS II) results in a disproportion between the production and consumption of electrons, resulting in alterations in quantum yield. These types of modifications in the chloroplast photochemistry in the plant leaves of salt- or drought-stressed plants result in a rakishness of excess light energy in the PS II and generate different free radicals such as O_2_^•−^, ^1^O_2_, H_2_O_2_, and OH^•^, which are potentially hazardous and create oxidative stress for plants [4]. Under HT stress, RuBisCO is able to generate H_2_O_2_ via oxygenase reactions [33]. On the other hand, under LT/cold stress, the solubility of a gas in plant cells increases. As a result, O_2_ concentration increases in cells and raises the threat of oxidative damages at LT, which leads to the amplified manufacture of O_2_^•−^, H_2_O_2_, ^1^O_2_, and OH^•^ [34]. Reduction of photosynthetic pigment content, inhibition of photochemistry efficiency or the uneven function of PS II, inhibition of biochemical metabolism, or enzymatic activities under LT stress might also be associated with excess ROS generation which is similar to other abiotic stresses [17,20]. Under flooding/waterlogging conditions, the photosynthetic ETC becomes over-reduced, causing the generation of several ROS, including H_2_O_2_, OH^•^, and ^1^O_2_ [35]. Normally, heavy metal (HM) stress affects the H_2_O oxidizing system of PS II because HMs are able to replace Ca^2+^ and Mn^2+^ ions in the PS II reaction center; thus, hindering the reaction of PS II results in the uncoupling of the electron transport in the chloroplast. The redox-active HMs such as Fe, Cu, Cr, V, and Co enable redox reactions in the cell. They are involved in the formation of OH^•^ from H_2_O_2_ via Haber–Weiss and Fenton reactions and initiate non-specific lipid peroxidation [36]. However, non-redox-active HM, such as Cd^2+^ is unable to generate ROS directly through Haber-Weiss reactions. Reactive oxygen species overproduction and the occurrence of oxidative stress in plants are the indirect consequence of cadmium (Cd) toxicity. The mechanisms include the interaction of Cd with the antioxidant system, the induction of NADPH (nicotinamide adenine dinucleotide phosphate) oxidase, and the disruption of the ETC as well as the metabolism of essential plant nutrients [37,38,39].

## 3. Methylglyoxal: An Unavoidable Foe for Plants

Methylglyoxal, a cytotoxic compound, is a byproduct of different metabolic activities of the organism. Chloroplast, mitochondrion, and cytosol are potential sources of MG [40,41]. The continuous production of MG carries on as a result of glycolysis [8,40,41]. In normal growth conditions, plant cells contain a small amount of MG; however, under different kinds of abiotic stress, MG production gradually increases [8,42,43,44,45]. Therefore, both ROS and MG become toxic for plants under different abiotic stresses because those stresses result in oxidative stress. The main deleterious effect of oxidative stress is the oxidation of cellular components such as lipid peroxidation, protein degradation, and DNA mutations [31]. To be more specific, MG, having ketone and aldehyde functional groups, reacts with deoxyguanosine residues of DNA to produce imidazopurinone MGdG isomers and reacts with guanidino groups of arginine to glycate protein, producing hydroimidazolone *N*(δ)-(5-hydro-5-methyl-4-imidazolon-2-yl)-ornithine (MG-H1) residues known as advanced glycation end products (AGEs) [46,47]. Subsequently, these AGEs cause protein inactivation and oxidative damage in major cell constituents. Therefore, plants, as well as other living organisms, have the glyoxalase system to protect DNA and protein by converting MG into d-lactate. However, under abiotic stress, MG concentration increases at a rate that is usually higher than the rate of detoxification by the glyoxalase system. A higher accumulation of MG results in the inhibition of germination and cell proliferation and causes the glycation of proteins, the disruption of the antioxidant defense system, and other metabolic dysfunctions [27,28,48,49]. Mankikar and Rangekar [50] found the inhibition of seed germination in a dose-dependent manner with the concentration of MG. Furthermore, MG inhibited the synthesis of protein and nucleic acid. Along with seed germination, root elongation was significantly reduced due to 1 and 10 mM MG and chlorosis occurred at 10 mM MG in *Arabidopsis* [28]. *Arabidopsis* seed germination was unaffected by MG at concentrations 0.1 and 1.0 mM, but seedling growth reduced considerably in both wild-type and d-LDH knock out lines (dldh1-1, d-ldh1-2) in a dose-dependent manner. The severe reduction in d-LDH knock out lines confirms d-lactate dehydrogenase involvement in MG metabolism [51]. Similarly, growth of both tomato and tobacco seedlings were retarded greatly by 1 mM MG [52]. In a recent study, Kaur et al. [40] showed that MG at concentrations of 5, 7.5, 10, 15, and 20 mM caused a reduction in both shoot and root length in a dose-dependent manner, and this result is coherent with previous research reports. One reason for this growth reduction in root and shoot may be the inhibition of photosynthesis by MG, as it hampers photosynthesis by inactivating the CO_2_-photoreduction by 17% [53].

## 4. Methylglyoxal Biosynthesis and Metabolism in Plants

**Figure 2:**
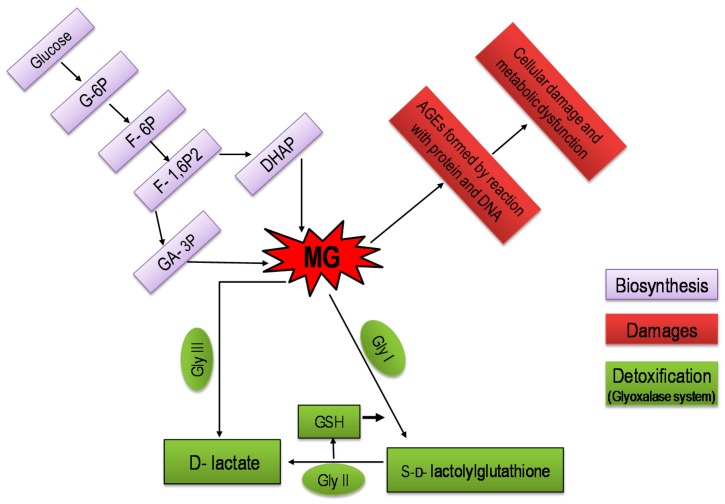
Methylglyoxal biosynthesis, damaging effects, and its detoxification through the glyoxalase system (modified from Kalapos [56] and Kaur et al. [48]) (G-6P, glucose 6-phosphate; F-6P, fructose 6-phosphate; F-1,6P2, fructose 1,6-bisphosphate; GA-3P, glyceraldehyde 3-phosphate; DHAP, dihydroxyacetone-phosphate; GSH, glutathione; Gly I, Glyoxalase I; Gly II, Glyoxalase II; Gly III, Glyoxalase III; AGEs, advanced glycation end products).

Methylglyoxal can be produced in living organisms through both enzymatic and non-enzymatic pathways. In enzymatic pathways, three enzymes can generate MG by catalyzing three different metabolites. For example, MG synthase catalyzes the reaction where dihydroxyacetone phosphate (DHAP) is converted to MG and inorganic phosphate, another enzyme called cytochrome P450 can also generate MG from acetone, and MG can similarly be produced from aminoacetone by amine oxidase enzyme. These three enzymes present in mammals, yeasts, and, microbes—surprisingly, but not in plants [48,54]. Unlike mammals, yeasts, and microbes, MG is produced in plants mainly by the non-enzymatic route from glyceraldehyde-3-phosphate (GAP), which is an intermediate of glycolysis and photosynthesis, and from DHAP (Figure 2) [48]. The mechanism of non-enzymatic MG formation was explained by Richard [55]. The formation of MG from triosephosphates occurs through β-elimination of the phosphoryl group from 1,2-enediolate of these trioses, and the rate of this non-enzymatic MG formation is 0.1 mM·day^−1^ [55]. However, it is suspected that other ways of MG formation may be possible in plants, including the metabolism of aminoacetone and acetone [48,56].

Methylglyoxal production is an unavoidable consequence of metabolism, even in normal physiological conditions in living organisms. The major route for MG detoxification is through the glyoxalase system, ubiquitously present in mammals, yeasts, bacteria, and plants [49,57]. The glyoxalase enzymes viz. Gly I and Gly II act coordinately to detoxify MG by converting it into a non-toxic product using GSH as a cofactor (Figure 2). However, Ghosh et al. [25] proposed a short route for MG detoxification, where Gly III can convert MG into d-lactate without using GSH. Along with glyoxalase systems, MG can be detoxified via some minor routes. For example, the enzymes involved in oxido-reductions can reduce MG to α-oxoaldehyde, as MG contains ketone and aldehyde as functional groups [56]. Therefore, some enzymes such as aldose/aldehyde reductase (ALR) or aldo-keto reductase (AKR) are considered to potentially detoxify MG. Hegedüs et al. [58] reported that transgenic tobacco overexpressing ALR reduced malondialdehyde (MDA) content and conferred tolerance to cold and Cd stress. In addition, transgenic tobacco overexpressing *OsAKR1*, an AKR gene, decreased MG and MDA content under methyl viologen (MV) and HT [59]. Though these studies suggest the role of ALRs and AKRs in the reduction of MG and MG-like aldehyde under stress, the mechanism of MG detoxification through minor routes are still unclear [60].

## 5. Glyoxalase System: The Eliminator of Methylglyoxal in Plants

The MG detoxifying glyoxalase pathway was first reported by two independent groups [61,62] in 1913. The existence of the glyoxalase system was reported in plants in the last decade of the previous century [63,64], which is located in cytosol and other organelles of chloroplast and mitochondria [43,46]. Two enzymes, Gly I and Gly II, and GSH act coordinately to eliminate MG ([65]; Figure 2). Due to the involvement of GSH, this MG elimination pathway is also known as a GSH-dependent glyoxalase pathway [48]. However, in the first step of the glyoxalase system, MG produces hemithioacetal via spontaneous reaction with GSH. Hemithioacetal is converted to SLG via 1,2-hydrogen transfer, which is catalyzed by the Gly I enzyme. In the second step, Gly II enzyme converts SLG to d-lactate by hydrolysis, which later confirms the regeneration of GSH [48,65]. Therefore, overexpression or higher activity of glyoxalase enzymes eliminate MG toxicity and confer stress tolerance [48]. Many reports have shown increased or overexpressed Gly I or Gly II as well as both reduced endogenous MG levels and conferred stress tolerance. Overexpression of the *Gly I* gene in transgenic tobacco showed better stress tolerance than the normal plant to MG and high salinity [66]. Under salt stress, transgenic rice has also shown better tolerance to high MG levels by overexpression of the *Gly II* gene [67]. Overexpression of both the *Gly I* and *Gly II* genes showed better stress tolerance in transgenic tobacco [68]. Increased activities of Gly I and Gly II also confer stress tolerance in rice and mung bean by reducing MG levels under abiotic stress conditions [21,69,70,71,72,73]. Recently, a GSH-independent glyoxalase enzyme named Gly III has been detected in plants that is capable of MG detoxification in a single step. In this step, MG converted to d-lactate by the Gly III enzyme without formation of SLG and/or involving GSH or any other cofactor [25].

## 6. Coordinated Actions of Glyoxalase and Antioxidant Defense System in Mitigating Oxidative Stress in Plants

**Figure 3:**
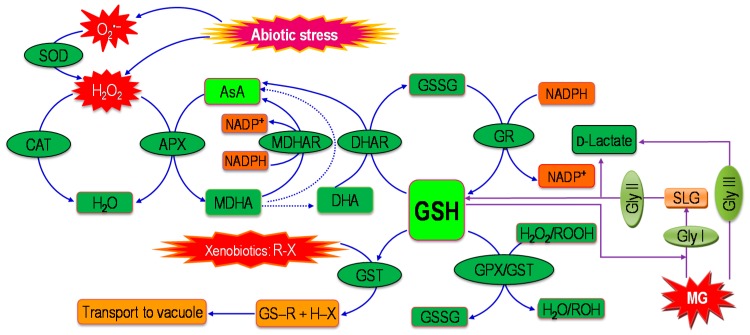
Coordinated actions of antioxidant defense and glyoxalase systems in eliminating the toxic ROS and MG under abiotic stress. Dotted lines denote non-enzymatic conversions. R may be an aliphatic, aromatic, or heterocyclic group; X may be a sulfate, nitrite, or halide group. (Ascorbic acid, AsA; glutathione, GSH; glutathione dissulfide, GSSG; superoxide dismutase, SOD; catalase, CAT; ascorbate peroxidase, APX; MDHA, monodehydroascorbate; monodehydroascorbate reductase, MDHAR; DHA, dehydroascorbate; dehydroascorbate reductase, DHAR; glutathione reductase, GR; glutathione peroxidase, GPX; glutathione *S*-transferase, GST; NADP, nicotinamide adenine dinucleotide phosphate; Gly I, glyoxalase I; Gly II, glyoxalase II; SLG, *S*-D-lactoylglutathione). (Adapted from Hasanuzzaman et al. [4].) Solid arrows indicate enzymatic reactions while dotted arrows indicate non-enzymatic reactions.

It is evident that MG production increased under abiotic stress, which plays a role in the over-formation of ROS generation. Under stress, MG increases ROS formation in plant cells directly due to the presence of MG. On the other hand, MG increases ROS formation in plant cells indirectly through the formation of advanced glycation end product (AGEs). Consequently, MG causes a higher formation of ROS, which plays a vital role in inducing oxidative stress [43,74,75]. As a result, elimination of MG can inhibit MG-induced ROS production. On the other hand, production of ROS is unavoidable, and plants are well equipped by their antioxidant defense system to detoxify overproduced ROS. Under environmental stress, ROS production has been shown to increase and is readily scavenged by the plant antioxidant defense system that directly mitigates oxidative stress [10]. There are several enzymatic and non-enzymatic antioxidants that lead to antioxidant defense and defend against oxidative stress [4] (Figure 3). In such cases, GSH plays a central role because it is used in the glyoxalase system by Gly I, which is again regenerated by the action of Gly II (Figure 3). As both antioxidant and glyoxalase systems are involved in ROS detoxification, the coordination of these systems can mitigate oxidative stress by reducing ROS production (Figure 3). Many studies showed the coordinated actions of antioxidant defense and glyoxalase systems in mitigating oxidative stress by detoxifying both ROS and MG, respectively. Exogenously applied phytoprotectants confer environmental stress tolerance by the coordinated action of the upregulated antioxidant defense and the glyoxalase systems in *Triticum aestivum* [13,16], *Brassica napus* [11,76], *Oryza sativa* [77,78,79,80,81], and *Vigna radiata* seedlings under abiotic stresses. These are discussed in the following sections.

## 7. Regulation of the Glyoxalase System and the Antioxidant Defense System in Plants under Abiotic Stress

### 7.1. Salinity

Under abiotic stress, the production of MG content increased drastically due to a higher rate of glycolysis, amino acid, and acetone metabolism, or other biochemical processes. Due to a higher production of MG, the glyoxalase system is regulated under various environmental stresses, including salinity [8,42,67,82]. However, many reports have shown that MG levels increase and glyoxalase enzymes upregulate or downregulate depending on salinity level, stress duration, and/or plant species (Table 1). The exposure of 300 mM NaCl has been shown to increase the MG level and Gly I activity after 24 h in *Cucurbita maxima* [57]. Hossain and Fujita [83] reported that salt stress caused ROS-induced oxidative stress in *V. radiata* seedlings with a concomitant decrease in the redox state of GSH. They also reported that exogenously applied proline (Pro) and glycinebetaine (GB) alleviated salt-induced oxidative stress by involving ROS and MG detoxification. Hossain et al. [84] also noted that the exposure of 150 mM NaCl regulated antioxidant defense and glyoxalase systems (increased Gly I and Gly II activity) in *B. campestris*, which is further upregulated by 5 h of heat shock and alleviated salt-induced damage through ROS and MG detoxification. On the other hand, Hasanuzzaman et al. [11,76,77] showed that increased salinity decreased Gly I and Gly II activity along with increased ROS production in *Triticum aestivum*, *B. napus,* and *O. sativa* seedlings (Table 1). They also showed that the exogenous application of phytoprotectants (nitric oxide, salicylic acid, Pro, and GB) increased ROS detoxification by upregulating antioxidant defense and glyoxalase (further stimulating Gly I and Gly II activities) systems and mitigating salt-induced oxidative stress. Mostofa et al. [78] reported that glyoxalase enzyme (Gly I and Gly II) activity and the level of oxidative stress increased with increasing salinity levels in rice seedlings (Table 1). Later, Mostofa et al. [79] showed that exposure to 150 mM NaCl disrupted glyoxalase (increased MG content and decreased Gly I activity) and antioxidant defense (increased ROS production) systems in *O. sativa* seedlings. Recently, Rahman et al. [80,81] showed that the exposure of 150 and 200 mM NaCl increased ROS production by disrupting ion homeostasis and antioxidant defense and glyoxalase (increased MG production) systems. They also noted that the exogenous application of Ca and Mn reduced ROS-induced oxidative damage by the coordinated action of nutrient homeostasis and antioxidant defense and glyoxalase systems (Table 1).

### 7.2. Drought

Drought stress indicates a deficit of water such that substantial damage to plant developmental processes results. Disrupted or reduced enzyme activities, loss of cell water content, or turgor are the primary effects of drought stress, causing reductions in cell division and expansion as well as in plant growth [22,86]. Like other stresses, drought also overproduced MG, interrupting the glyoxalase system as documented in several plant studies (Table 2) [14,17,18,19,20,21,22,23,24].

Drought stress augmented the activity of Gly I but diminished the activity of Gly II in rapeseed seedlings. Via drought treatment, increases in GSH and GSSG content and decreases in GSH/GSSG ratios have been demonstrated. The level of AsA has been shown to increase only under mild stress (induced by 10% PEG, compared to severe drought stress induced by 20%). The MDHAR and GR activities have increased only under mild stress (10% PEG). The activities of DHAR, GST, and GPX have increased, but CAT activity has decreased at both levels of stress. Disruption of antioxidant defense and glyoxalase system via drought treatments was shown to result in a sharp increase in H_2_O_2_ and lipid peroxidation, which are indicators of oxidative stress [14]. Mung bean (*V. radiata* L. cv. Binamoog-1) seedlings were subjected to drought stress (induced by 25% polyethylene glycol 6000, PEG) for 24 and 48 h. Drought stress increased the level of MG content with a concomitant increase in GSH content and activities of Gly I and Gly II. Drought-affected seedlings faced oxidative stress, which is clear from the high increase in H_2_O_2_ and O_2_^•−^ content and their spots in the leaves (detected by histochemical staining), which was due to a disrupted antioxidant defense system (decreased AsA content, increased GSH and GSSG contents, decreased GSH/GSSG ratio, and decreased MDHAR, DHAR, and CAT activities). Some other physiological attributes of mung bean seedlings such as leaf chl content, leaf succulence, and relative water content (RWC) have been shown to decrease, and proline (Pro) content has been shown to increase due to drought [19]. *Oryza sativa* L. cv. IR64 overexpressed *OsDJ-1C*, which enhanced MG detoxification and the formation of d-lactate. In this conversion of d-lactate from MG, the activity of the Gly III enzyme was involved in demonstrating the existence of functional GLY III as a shorter route for MG detoxification [25]. Osmotic stress also upregulated the Gly I activity in tomato [87]. MG increased significantly in tobacco plants in response to drought [88]. Tomato (encoding gene for Gly I) plants showed enhanced Gly I activity 2–3-fold in all cell types of roots, stems, and leaves, especially in phloem sieve elements, under water deficit stress [87]. The *Glyoxalase I* gene was cloned and characterized from *B. juncea*. The expression of *Gly I* was upregulated in response to water stress. The level of transcript, protein, and specific activity of Gly I was markedly enhanced under water stress [66]. In another study, *V. radiata* seedlings were subjected to drought stress (induced by 5% PEG, 48 h), their performance was compared with control seedlings. Exogenous drought treatment caused endogenous drought stress, which is reflected in a decreased leaf RWC, water saturation deficit, water retention capacity, and increased Pro. According to the investigation, drought-affected seedlings faced oxidative damage, which was exhibited by a breakdown of chl and increased lipid peroxidation. The reason behind this was that overproduction of ROS including H_2_O_2_ and O_2_^•−^, an increase in LOX activity, a disruption of the antioxidant defense system (decreased content of AsA, AsA/DHA and GSH/GSSG ratios, and activities of CAT, APX, MDHAR, DHAR, and GR) and obviously the overproduction of toxic MG, which is not only directly responsible for oxidative damage but also responsible for ROS production that causes oxidative damage. The increase in toxic MG was due to the modulation of glyoxalase system components including Gly I and Gly II activities, the content of GSH and GSSG, and the ratio of GSH/GSSG [22]. Aldose/aldehyde reductase is cytosolic NADPH-dependent oxidoreductase, catalyzing the reduction of a variety of aldehydes and carbonyls. According to Hideg et al. [89], under drought stress, transgenic tobacco (compared to control) showed higher ALR activity, which was highly correlated to reduced H_2_O_2_ and OH^•^ radical production, as well as the production of thiobarbituric acid reactive species (lipid peroxidation products), all of which indicate a reduction in oxidative damage. Transformed plants were more tolerant and exhibited a reduced loss of photosynthetic function. In a recent study, higher ALR activity might have roles in reducing MG content, which further decreased ROS production, but this needs further investigation [89]. A similar report was observed in another previous study [90]. The recombinant alfalfa had been developed for ALR. A reduction in the generation of lipid peroxidation-derived reactive aldehydes in these transformed plants has been documented over a long period of water deficiency, which also showed improved recovery after rehydration [90].

### 7.3. Toxic Metals/Metalloids

Toxic metal stress drastically amplifies the MG level of plant cells and creates oxidative stress [21,23,69,91,92]. Many recent studies have confirmed that, under metal stress, the level of MG increases without a proper detoxification process of the glyoxalase system (Table 3). Sometimes, the individual enzymes of the glyoxalase system, Gly I and Gly II, or both, increase in the primary stage of metal stress. However, in most cases, with an increase in the duration of stress, the activity of glyoxalase enzymes decreases. Therefore, the increase or decrease of glyoxalase enzymes depends on stress intensity and duration. However, many researchers have used different kinds of plant protectants against metal stresses and found that these types of protectants upregulated the enzymes of the glyoxalase system and defended toxic MG or MG-induced oxidative stress. Hossain et al. [57] observed that 1 mM CdCl_2_ stress for 24 h in *Cucurbita maxima* increased MG and upregulated the activity of the Gly I enzyme. This upregulation of Gly I activity may be due to a short duration of stress. However, in *V. radiata*, 1 mM CdCl_2_ stress for 48 h, slightly increased Gly I activity but decreased Gly II activity as well as increased accumulation of MG [93]. However, significant reduction of this MG enhancement was observed after using exogenous protectant (5 mM Pro or 5 mM GB). Hasanuzzaman et al. [94] carried out an experiment with rapeseed plant under 0.5 and 1.0 mM CdCl_2_ stress and demonstrated that Gly I activity significantly decreased by 18% and 35% with 0.5 and 1.0 mM CdCl_2_, respectively and Gly II activity significantly decreased by 20% and 32% with 0.5 and 1.0 mM CdCl_2_, respectively. However, after pretreatment of Na_2_SeO_4_, they observed both enzymes of the glyoxalase system significantly upregulated. Later on, Hasanuzzaman and Fujita [16] recorded the same trend in wheat plants under arsenic (As) stress. Both Gly I and Gly II activities decreased under As stress in wheat plants, which upregulated after using SNP as exogenous protectant. However, rice plants under 150 µM CuSO_4_ stress for 48 h significantly increased Gly I and Gly II activities in both leaves and roots [95]. Both Gly I and Gly II activities further increased in roots after pretreatment of SA; however, in leaves, Gly I activity increased further by 50%, and Gly II activity remain unchanged. On the other hand, Mostofa et al. [96] reported that Gly I and Gly II activities increased significantly in rice plants exposed to 100 μM CuSO_4_ for 48 h. However, no significant differences were observed in glyoxalase system enzymes after SNP treatment; even GSH treatment decreased enzyme activity. Mostofa et al. [79] conducted an experiment with rice plants (BRRI dhan29) under 100 µM CuSO_4_ stress for 4 and 7 days and found activity of the glyoxalase system damaged in a time-dependent manner. They recorded that MG increased by 106% and 156% after 4 and 7 days of stress, respectively; Gly I activity increased by 22% after 4 days of stress treatment and decreased by 25% after 7 days of stress and Gly II activity increased by 47% after 4 days of stress, but the activity returned to the level in the control after 7 days of stress. However, pre-treatment with 10 mM Tre for 48 h upregulated the enzymes of the glyoxalase system and reduced the production of toxic MG. Rahman et al. [69] observed that rice seedlings under 0.5 and 1 mM Na_2_HAsO_4_ stress for 5 days decreased Gly I activity by 9% and 17%, respectively, but increased Gly II activity as well as MG content. However, combined treatment of 10 mM CaCl_2_ and Na_2_HAsO_4_ stress showed higher Gly I and Gly II activities. MG content also decreased by 22% and 25% at 0.5 and 1 mM Na_2_HAsO_4_-treated rice seedlings, respectively. Both 0.25 and 0.5 mM CdCl_2_ stress for 72 h in rice plants increased MG content with the decline of Gly I and Gly II activities [91]. However, cotreatment of 2.5 mM CaCl_2_ with stress decreased MG content by 31% and 24% at 0.25 and 0.5 mM Cd-treated seedlings, respectively, increased Gly I activity by 35% and 31% at 0.25 and 0.5 mM Cd-treated seedlings, respectively, and increased Gly II activity by 23% and 53% with 0.25 and 0.5 mM Cd exposure, respectively. Recently, Rahman et al. [92] reported that rice plants under Cd stress showed high MG content due to inefficient activity of enzymes of the glyoxalase system, but co-application of 0.3 mM MnSO_4_ upregulated the Gly I and Gly II enzymes, which lessened the MG level. In *V. radiata*, 1.5 mM CdCl_2_ stress for 48 h increased MG content by 132% with increased Gly I and decreased Gly II activities [21]. On the other hand, use of exogenous protectant increased the activity of Gly II enzyme and decreased the cytotoxic MG level. Very recently, Nahar et al. [22] observed that MG content increased by 77% and 177% under 1.0 and 1.5 mM CdCl_2_, respectively, in mung bean plants with increased Gly I and decreased Gly II activities. However, pretreatment of 0.25 mM spermine (Spm) for 24 h in mung bean plant reduced the MG level with the slight increase in Gly I and marked increase in Gly II activities. The above findings indicate that the increase in MG content is a common response of plants to a metal stress, and that the glyoxalase system primarily tries to detoxify the MG but becomes unable under severe stress. However, the use of diverse exogenous protectants can overcome these problems at certain levels by maintaining elevated Gly I and Gly II activities, thus creating the possibility of upregulating the GSH level and the GSH/GSSG ratio via the glyoxalase system. The high GSH levels assist in the synthesis of the phytochelatin and these questration of the heavy metal phytochelatin complex into the vacuole [21,23,93,94].

### 7.4. Extreme Temperatures

High temperatures beyond plants’ tolerance levels lead to physiological disorder and catastrophic loss of crop productivity (Table 4) [5,6]. High temperature stress results in malfunctioning of PS II and decreases electron transport efficiency, which are the reasons for the overproduction of ROS in plants [97]. Disrupting the activities of the glyoxalase system, HT enhances the production of cytotoxic MG [18,22]. Low temperature also induces overproduction of ROS and MG because of the disruption of antioxidant defense and glyoxalase systems, respectively which is same as effects of other abiotic stresses [17,20]. Understanding the mechanism of damage by HT or LT is fundamental for the development of tolerant plant species.

*Ficus concinna* seedlings were grown under HT stress of 35 °C (considered as moderate HT stress) and 40 °C (considered as severe stress) and their performance were compared with the control seedlings (grown under 28 °C). The activity of Gly II increased under both levels of HT stresses. However, Gly I activity increased only at moderate HT stress. High temperature stress showed damaging effects by increasing the MG level and inducing oxidative stress through the generation of ROS (O_2_^•−^ and H_2_O_2_) and the increase in MDA and MG content. The reductions in chl levels and relative water content were noticed in HT-affected seedlings [98]. A coordinated induction of glyoxalase and antioxidant defense systems has been documented in mung bean seedlings under HT stress. High temperature stress (40 °C, 2 days) resulted in a high increase in MG and increased Gly I activity, but decreased Gly II activity, increased GSH content, and a decreased GSH/GSSG ratio in mung bean seedlings. Increased H_2_O_2_ and O_2_^•−^ generation and decreased AsA content and AsA/DHA ratios have been noticed in HT-affected seedlings. Differential modulations of enzymes of antioxidant defense system were observed under HT stress, and these modulations include the reduction in CAT, MDHAR, and DHAR activities and increased APX, GR, GPX, and GST activities. High temperatures also decreased water content, increased Pro content, destroyed chl pigment, and decreased the seedlings’ vigor and biomass accumulation [22]. The leaf discs from transgenic tobacco plants subjected to 44 °C exhibited higher AKR activity and was responsible for accumulating a lower amount of MG in their leaves, compared to the wild-type plants with either a presence or absence of HT stress, which improved HT stress tolerance [59]. Overexpression of *OsglyII* resulted in the rapid accumulation of Gly II in rice under HT (45 °C) and LT stress (4 °C) [99]. Temperature shock was reported to transduce signal through Ca^2+^, which binds to specific target proteins, such as kinases, which in turn activated Gly I [100]. Low temperature stress (6 °C) increased MG content, H_2_O_2_ content, and lipid peroxidation and decreased Gly II activity, water content, and growth in mung bean seedlings. The Gly I activity remained unaltered, but Gly II activity decreased, which was the cause of the increase in MG content in both 2 and 3 days of LT stresses [17]. High temperature treatment (38 °C, 24 and 48 h) increased Gly I and Gly II activities, MDA, and H_2_O_2_ levels, but decreased the chl content in *T. aestivum* L. cv. Pradip. Modulation of antioxidant system components have been demonstrated in HT-affected wheat seedlings. Ascorbate content decreased, GSH and GSSG content increased, the GSH/GSSG ratio decreased, and the activities of APX, GR, GPX, and GST increased upon HT exposure [13]. Most recently, the enzyme Gly III’s efficiency in depleting MG has been shown in a shorter pathway. *Oryza sativa* L. cv. IR64 overexpressing *OsDJ-1C* was exposed to 4 and 42 °C for 8 h. Increased activity of Gly III enzyme has been documented with a decreased level of MG and the simultaneous formation of d-lactate, indicating the role of *OsDJ-1C* as a GLY III enzyme converting MG directly into d-lactate in a GSH-independent manner [25].

## 8. Role of Methylglyoxal as a Signaling Molecule

**Figure 4:**
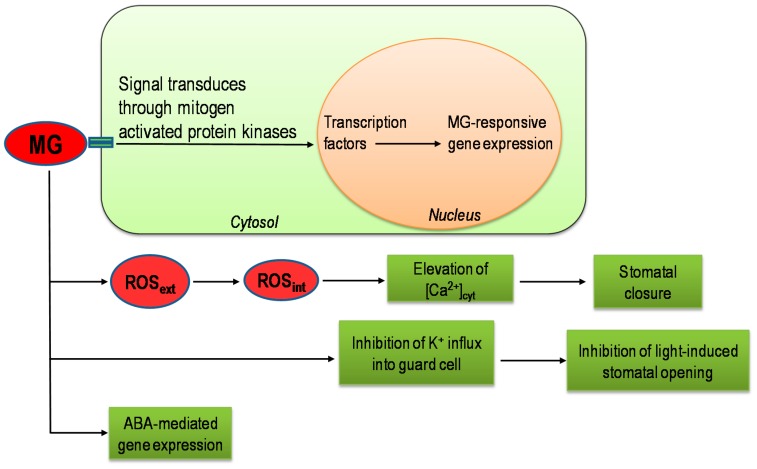
MG signaling pathways in plants (Modified from Hoque et al. [49] and Kaur et al. [40,41]) (ROS_ext_, extracellular ROS; ROS_int_, Intracellular ROS; [Ca^2+^]_cyt_, cytosolic Ca^2+^).

At normal growing conditions, the basal level of MG in plants is 30–75 µM [8,42,82]. However, under stress, the amount of MG increases. Therefore, it is possible that MG may take part in signaling pathways in plants. The signaling role of MG is explained in a study by Hoque et al. [27]. They reported that MG can induce stomatal closure by modulating ROS production and cytosolic-free calcium concentration in the *Arabidopsis* leaf guard cells. MG-induced stomatal closure without involving endogenous abscisic acid (ABA) or endogenous methyl jasmonate is worth noting. In another study, Hoque et al. [28] reported that the MG-induced stomatal closure in *Arabidopsis* is due to the inhibition of K^+^ influx into the guard cells. Furthermore, the involvement of MG in the regulation of ABA-induced signaling pathways was shown by Hoque et al. [101]. In their study, MG induced the expression of RD (responsive to dehydration) genes (*RD29A* and *RD29B*) in *Arabidopsis*, whereas an *aba2-2* mutant (ABA-deficient mutant) showed no expression of *RD29B* and *RAB18* (responsive to ABA gene) genes. Recently, microarray analysis of rice treated with MG showed the upregulation or downregulation of genes involved in signal transduction and abiotic and biotic stress responses. Exogenous MG affected the genes that control the complex signal transduction pathway. Signals are transmitted from cytosol to nucleus through phosphorylation or dephosphorylation of protein kinases. Therefore, a number of transcription regulatory proteins such as *DREB*, *MYB*, *NAC*, *WRKY*, and *AP2* domain-containing proteins, were modified due to MG exposure. Most of the transcription factors that are altered due to MG are known to play role in biotic and abiotic stresses in plants. Thus, there may be a cross-talk between MG-responsive and stress-responsive signal transduction pathways in plants. In addition to plants, MG also plays a role in signal transduction in animals, bacteria, and yeast [40,41]. Furthermore, it is worth noting that the MG responsive element, 7–8 bp long conserved elements in the promoter region of the genes, has been identified in rice by microarray analysis [40]. Though the possible pathways of MG-induced signal transduction have been determined and are shown in Figure 4, the concrete mechanism is yet to be explored in plants.

## 9. Genetic Manipulation in Enhancing Glyoxalase Pathway in Plants

Engineering of the glyoxalase pathway has been documented to enhance abiotic stress tolerance in different plant species (Table 5). The *GLX2-1* gene expression is upregulated in wild *A. thaliana* under salt, anoxia, and excess l-threonine stresses, indicating the essential roles of glyoxalase system enzymes under stress conditions [102]. Transgenic tobacco underexpressing Gly I accumulated significantly higher content of MG and showed inhibition of seed germination under different abiotic stress conditions (salinity, 200 mM; cold, 4 °C; drought stress, withholding watering). In contrast, in Gly I overexpressing (*NtSgly I*) transgenic tobacco, MG content did not increase much in response to different abiotic stresses, compared to the untransformed plants. Moreover, the supplementation of exogenous GSH reduced MG levels in both untransformed and transgenic plants [42]. Overexpression of the *gly I* gene resulted in a higher activity of the Gly I enzyme in transgenic *V. mungo* that improved the ability to withstand salt stress. The transgenic line also showed improved germination and growth under salt stress [103]. The cDNA encoding Gly I was cloned and characterized from *B. juncea*, and transgenic tobacco plants overexpressing Gly I were developed. The transgenic tobacco plants were tolerant to MG and high salt. The degree of Gly I expression was positively correlated to various levels of salt stresses, indicating the pivotal role of Gly I in ensuring salt stress tolerance [66]. Overexpression of *TcGLX1* increased levels of Gly I, decreased metal accumulation, and improved root growth under zinc stress. Transgenic *Thlaspi caerulescens* also showed similar tolerance response to Cd and lead [104]. Transgenic *T. aestivum* overexpressing *TaGly I* was exposed to NaCl and ZnCl_2_ stresses. However, transgenic tobacco showed higher tolerance to ZnCl_2_ stress, compared to the control [105]. Transgenic *Carrizo citrange* rootstocks overexpressing Gly I (*BjGlyI*) and Gly II genes (*PgGlyII*) were examined and compared with the wild type for its salt (75 mM NaCl) stress tolerance capacity. The wild type showed yellowing and marginal burn in lower leaves, whereas the phenotypic performance of the transgenic plants was better, compared to the wild type. The dry weight of the root, shoot, and whole plant was higher in the transgenic plant that those of the wild plant. The reason behind the better performance of the transgenic plant was due to a smaller accumulation of Na^+^ and Cl^−^ ions, compared to the wild-type plant. Therefore, the heterologous expression of glyoxalase system genes improved salt stress tolerance in *Carrizo citrange* [106]. Sugar beet M14 line encoding M14 glyoxalase I is an interspecific hybrid between a wild species *Beta corolliflora* Zoss and a cultivated species *B. vulgaris*. Upon expression of M14 *glyoxalase I*, the transgenic sugar beet showed improved tolerance to MG, salt, mannitol, and H_2_O_2_ stresses, compared to the wild type [107]. *Oryza sativa* L. cv. IR64 overexpressed *OsDJ-1C* has been reported as a Gly III enzyme. Methylglyoxal is directly converted into d-lactate in a GSH-independent manner due to Gly III activity. Thus, overexpression of *OsDJ-1C* increased the formation of d-lactate as a result of the depletion of MG, where the activity of Gly III was involved [25]. The expression of *Bj glyII* in *B. juncea* was upregulated by salinity, heavy metal stress, and ABA, where the activity of Gly II enzyme also increased [108]. Transcript profusion of *GLY I* and *GLY II* genes in rice was studied in response to various abiotic stresses including salt, drought, osmotic, cold, heat, oxidative, genotoxic, wounding, and UV/B stress and at different developmental stages [109]. Overexpression of *OsGLYI-11.2* in tobacco plants reduced MG content and decreased the Na^+^/K^+^ ratio and the maintenance of reduced glutathione levels under 200 mM NaCl or 1 mM MG or 5 mM H_2_O_2_ stresses [110]. Upregulation of the Gly I enzyme activity had been reported in *glyII*, overexpressing transgenic tobacco plants that showed reduced Na^+^ sequestration in the young leaves [68]. Overexpression of *OsglyII* showed higher tolerance to toxic concentrations of MG and NaCl in rice seedlings. The activity of Gly II increased under toxic concentrations of MG and NaCl stresses. Transgenic plants showed improved growth, increased shoot and root K^+^ content, and better ion balance (ratio of Na^+^/K^+^) under salt stress [67]. Again, transgenic tobacco plants overexpressing the genes for Gly I and Gly II enzymes were exposed to 5 mM ZnCl_2_ for 24 h. These tobacco plants showed a lower level of toxic Zn accumulation, and the reason behind this was the maintenance of the level of phytochelatins and glutathione homeostasis. Transgenic plants also showed better growth and flowering behavior, and they set normal viable seeds, better yield, MG accumulation, and less lipid peroxidation exposed to toxic level of Zn [67].

## 10. Conclusions and Outlook

In this review, we accommodate information from existing research findings and available reviews regarding the glyoxalase and antioxidant defense systems, and their pivotal functions in diminishing oxidative stress and cytotoxic effects on plants under different abiotic stresses. However, an array of laps and gaps remain behind these. Thus, questions arise on different issues. Is there any direct interaction between the glyoxalase and antioxidant defense systems since both of these systems utilize GSH-dependent pathways to detoxify ROS and MG, respectively [11,12,14,16,18,19,20,25,26]? Methylglyoxal catalyzes the photoreduction of O_2_ to O_2_^•−^ at photosystem I, and ROS production also has other pathways; is there any interaction among these pathways [26]? Very few reports indicate the signaling function of ROS and MG in plants [27,28,29]. However, the glyoxalase system has been studied widely in animal systems. Glyoxalase I and II have been purified and characterized from some plant species. Moreover, a very recent report explored the presence of Gly III proteins in plants, which performs the function of direct conversion of MG to d-lactate. Identification of this shorter route for MG detoxification is a signpost in the field of glyoxalase system study [25]. Higher ALR activity under stress conditions might have roles in reducing MG content, which further decreased ROS production, but it requires further investigation [89]. Upregulation of glyoxalase enzymes has been documented with the phytohormone accumulation together with rapid cell growth [87,111,112]. In plants, the roles of the glyoxalase system are not well defined. Moreover, exploration of the biological implication of this pathway under stress is just beginning. Therefore, study of the glyoxalase system in correlation with the antioxidant defense systems demands insightful research.

## Figures and Tables

**Table 1:** Regulation of the glyoxalase system and associated antioxidant defense system in plants under salt stress.

Plant Species	Stress (Dose and Duration)	Changes in Glyoxalase and Antioxidant Defense Systems	Protectants	Changes in Glyoxalase and Antioxidant Defense Systems after Protection	Reference
*V. radiata*	300 mM NaCl, 24 and 48 h	Increased Gly I activity Gly II activity increased to 1.5- and 1.2-fold after 24 and 48 h, respectively MDA and H_2_O_2_ content increased GSH and GSSG contents increased Increased GPX and GST activities	15 mM Pro, 15 mM GB	Further increase in Gly I activity by 12% and 17% after 24 and 48 h, respectively Further increase- of Gly II activity Increased GSH content Increased GPX, GST and GR activities Decreased MDA and H_2_O_2_ contents	[83]
*B. campestris*	150 mM NaCl, 48 h	Increased Gly I activity Gly II activity decreased by 18% Increased AsA and GSH contents Increased MDA and H_2_O_2_ contents Upregulated APX, MDHAR, DHAR and GR activities	Heat shock (42 °C), 5 h	Further increase in Gly I activity by 19% Increased Gly II activity by 22% Further increase in AsA and GSH levels and activities of APX, DHAR, GR and GPX	[84]
*B. napus*	100 and 200 mM NaCl, 48 h	Decreased Gly I activity by 21% and 33% with 100 and 200 mM NaCl, respectively Decreased Gly II activity by 30% and 37% with 100 and 200 mM NaCl, respectively Decreased AsA content Increased GSH and GSSG contents Increased production of H_2_O_2_ and lipid peroxidation Increased APX and GR activities Decreased MDHAR, DHAR and CAT activities	100 μM SA	Increased Gly I activity Gly II activity increased further by 31% and 37% with 100 and 200 mM NaCl, respectively Decreased ROS production and lipid peroxidation Further increase in ASA and GSH contents Upregulated APX, MDHAR, DHAR, GR and CAT activities	[76]
*T. aestivum*	150 and 300 mM NaCl, 4 days	Gly I activity decreased by 12% and 26% with 150 and 300 mM NaCl, respectively Decreased Gly II activity Decreased AsA content Increased GSH and GSSG contents Increased ROS production and lipid peroxidation with increasing salinity	1 mM sodium nitroprusside (SNP), 24 h pretreatment	Gly I activity further increased by 26% and 25% with 150 and 300 mM NaCl, respectively Increased Gly II activity Decreased ROS production and lipid peroxidation Increased ASA and GSH contents Increased MDHAR, DHAR and GR activities	[12]
*O. sativa*	150 and 300 mM NaCl	Gly I activity decreased in sensitive cultivar and increased in tolerant cultivar Decreased chlorophyll (chl) content Increased H_2_O_2_ production and lipid peroxidation Increased Pro content	5 mM Pro and 5 mM GB	Increased Gly I and Gly II activities Increased chl content Decreased ROS production and lipid peroxidation Upregulated non-enzymatic and enzymatic antioxidants	[77]
*O. sativa*	150 and 250 mM NaCl, 72 h	Increased Gly I and Gly II activities with increasing salt stress Increased ROS production and MDA content Decreased AsA content and increased GSH content Increased SOD, GPX, APX, DHAR and GR activities Decreased CAT and GST activities	10 mM Trehalose (Tre)	Further increase in Gly I activity Further increase in Gly II activity Increased SOD, CAT, MDHAR, DHAR and GR activities Decreased ROS production, lipid peroxidation lipoxygenase (LOX) activity and MDA content	[85]
*O. sativa*	150 mM NaCl, 4 days	Increased MG content by 58% Gly I activity increased by 12% Decreased chl content Increased ROS production Increased lipid peroxidation	50 μM H_2_S	Decreased MG content Increased Gly I and Gly II activities Increased chl content Decreased Pro content Decreased ROS production and lipid peroxidation	[78]
*O. sativa*	200 mM NaCl, 3 days	Increased MG content by 44% Increased Gly I and Gly II activities by 21% and 29%, respectively Increased ROS (H_2_O_2_, O_2_^•−^) production and lipid peroxidation (MDA content, LOX activity) Decreased AsA content and increased GSH content	2 mM CaCl_2_	Decreased MG content Further increased Gly I and Gly II activities by 24% and 20% respectively Increased ROS and MG detoxification	[80]
*O. sativa*	150 mM NaCl, 3 and 6 days	Increased MG content with increasing stress duration Increased Gly I and Gly II activities with increasing stress duration Increased ROS production Increased MDA content Decreased AsA content and increased DHA, GSH and GSSG contents	0.5 mM MnSO_4_	Decreased MG content Further increased Gly I and Gly II activities Increased ROS and MG detoxification Increased chl content Decreased osmotic stress (decreased Pro content, osmotic potential)	[81]

**Table 2:** Regulation of the glyoxalase system and associated antioxidant defense system in plants under drought stress.

Plant Species	Stress (Dose and Duration)	Changes in Glyoxalase and Antioxidant Defense Systems	Protectants	Changes in Glyoxalase and Antioxidant Defense Systems after Protection	Reference
*V. radiata*	5% PEG-6000, 48 h	Increased MG Increased Gly I activity but decreased Gly II activity Increased GSSG content, and ratio of GSH/GSSG Increased ROS generation and oxidative damage	0.2 mM Spermidine (Spd)	Increased Gly II activity Increased GSH and GSSG contents Reduced MG level Reduced ROS production including H_2_O_2_ and O_2_^•−^ as well as lipid peroxidation	[22]
*V. radiata*	25% PEG-6000, 24 and 48 h	Increased MG content Decreased activity of Gly II Increased H_2_O_2_ and O_2_^•−^ contents and lipid peroxidation	1 mM GSH	Increased activities of Gly I and Gly II Decreased MG content Decreased H_2_O_2_ and O_2_^•−^ contents and lipid peroxidation	[19]
*B. juncea*	Water deficit stress	Upregulation of Gly I activity Increased MG content	-	-	[66]
*S. lycopersicum*	Water deficit stress created by mannitol	Tomato (encoding gene for Gly I) upregulated Gly I activity by 2–3-fold in all cell types of roots, stems, and leaves especially in phloem sieve elements	-	-	[87]
*B. napus*	10% and 20% PEG-6000, 48 h	Gly I activity increased but Gly II activity decreased The content of GSH and GSSG increased, GSH/GSSG ratio decreased H_2_O_2_ and MDA contents increased	25 μM Na_2_SeO_4_	Increased activities of Gly I, and Gly II Increase in GSH content and GSH/GSSG ratio Reduction of ROS generation and oxidative damage	[14]
*O. sativa*	Desiccation, 8 h	Overexpression of OsDJ-1C increased Gly III activity, decreased MG content, increased formation of d-lactate in GSH-dependent manner	-	-	[25]

**Table 3:** Regulation of the glyoxalase system and the associated antioxidant defense system in plants exposed to toxic metals/metalloids.

Plant Species	Stress (Dose and Duration)	Changes in Glyoxalase and Antioxidant Defense Systems	Protectants	Changes in Glyoxalase and Antioxidant Defense Systems after Protection	Reference
*V. radiata*	1 mM CdCl_2_, 48 h	Slightly increased activity of Gly I Decreased Gly II activity Higher accumulation of MG	5 mM Pro or GB, 48 h	Further increase in Gly I activity Increased Gly II activity Lower oxidative damage due to higher MG detoxification	[93]
*B. napus*	0.5 and 1.0 mM CdCl_2_, 48 h	Gly I activity decreased by 18% and 35% at 0.5 and 1.0 mM CdCl_2_, respectively Gly II activity decreased by 20% and 32% at 0.5 and 1.0 mM CdCl_2_, respectively	Seed pretreatment; 50 and 100 μM Na_2_SeO_4_, 24 h	Further increase in Gly I activity Gly II activity increased	[94]
*T. aestivum*	0.25 and 0.5 mM Na_2_HAsO_4_·7H_2_O, 72 h	Decreased Gly I activity by 34% and 44% at 0.25 and 0.5 mM of As, respectively Decreased Gly II activity by 29% only upon 0.5 mM As Insufficient MG detoxification	0.25 mM SNP, 72 h	Increased Gly I and Gly II activities Efficient MG detoxification	[16]
*O. sativa*	150 µM CuSO_4_, 48 h	Enhanced Gly I activity Gly II activity increased by 24% in leaves and 30% in roots	Pretratment, 100 µM SA, 24 h	Further enhancement of Gly I activities in roots and leaves Enhanced Gly II activities by 50% in roots but remain statistically similar in leaves	[95]
*O. sativa*	100 μM CuSO_4_, 48 h	Increased Gly I and Gly II activities	200 μM SNP or 200 μM GSH, 48 h	Decreased Gly I and Gly II activities Increased GSH content	[96]
*O. sativa*	100 µM CuSO_4_, 4 and 7 days	Gly I activity increased by 22% after 4 days stress and decreased by 25% after 7 days stress Gly II activity increased by 47% after 4 days stress, but the activity returned to the level in control after Day 7 Increased MG level by 106% and 156% after 4 and 7 days stress, respectively	Pretreatment, 10 mM Tre, 48 h	Gly I activity increased at both days of Cu stress Gly II activity did not increase significantly at Day 4, but increased significantly at Day 7 Decreased MG level by 27% and 35% at 4 and 7 days stress, respectively	[79]
*O. sativa*	0.5 and 1 mM Na_2_HAsO_4_, 5 days	Decrease in Gly I activity by 9% and 17% at 0.5 and 1 mM As, respectively Increased Gly II activity MG content increased with dose-dependent manner	10 mM CaCl_2_ (Ca), 5 days	Higher Gly I activity Increased Gly II activity by 23% and 31% at 0.5 and 1 mM As treated seedlings MG content decreased by 22% and 25% at 0.5 and 1 mM As treated rice seedlings, respectively	[69]
*O. sativa*	0.25 and 0.5 mM CdCl_2_, 72 h	Reduced Gly I activity Declined Gly II activity Increased MG content with dose-dependent manner	2.5 mM CaCl_2_, 72 h	Increased Gly I activity by 35% and 31% at 0.25 and 0.5 mM CdCl_2_, respectively Increased Gly II activity by 23% and 53% with 0.25 and 0.5 mM CdCl_2_, respectively Decreased MG content by 31% and 24% at 0.25 and 0.5 mM CdCl_2_, respectively	[91]
*O. sativa*	0.3 mM CdCl_2_, 72 h	Increased Gly I activity Decreased Gly II activity Increased MG content	0.3 mM MnSO_4_, 72 h	Decreased the MG content Increased the Gly I activity Further increase in Gly II activity	[92]
*V. radiata*	1.5 mM CdCl_2_, 48 h	Increased Gly I activity Decreased Gly II activity Increased MG production by 132%	Pretreatment, 0.2 mM Put and 1 mM SNP, 24 h	Increased Gly II activity Decreased MG content	[21]
*V. radiata*	CdCl_2_, 1.0 and 1.5 mM	Increased Gly I activity Decreased Gly II activity Increased MG content by 77% and 177% under 1.0 and 1.5 mM CdCl_2_, respectively	Pretreatment, 0.25 mM Spm, 24 h	Slight increase in Gly I activity Markedly increased Gly II activity Reduced MG content	[23]

**Table 4:** Regulation of the glyoxalase system and associated antioxidant defense system in plants under temperature stress.

Plant Species	Extent of Temperature Stress	Changes in Glyoxalase and Antioxidant Defense Systems	Protectants	Changes in Glyoxalase and Antioxidant Defense Systems after Protection	Reference
*F. concinna*	35 and 40 °C, 48 h	Increased MG content and Gly II activity but decreased Gly I activity	0.25 µM 24-epibrassinolide (EBR)	Increased activities of Gly I and Gly II Decreased the levels of ROS, MDA and MG	[98]
*O. sativa*	45 °C, 15–120 min	Overexpression of *OsglyII* resulted in rapid accumulation of Gly II	-	-	[99]
*O. sativa*	42 °C, 8 h	Overexpressing *OsDJ-1C* the Gly III activity had been increased, decreased MG content, increased formation of d-lactate in GSH-dependent manner	-	-	[25]
*V. radiata*	40 °C, 2 days	Overproduction of MG Increased Gly I activity, decreased Gly II activity Increased GSH content, decreased GSH/GSSG ratio, increased H_2_O_2_ and O_2_^•−^ generation	Spermine (Spm, 0.2 mM)	Reduced MG content Increased Gly I and Gly II activities Increased GSH content and GSH/GSSG ratio Decreased H_2_O_2_ and O_2_^•−^ production	[22]
*T. aestivum*	38 °C, 24 and 48 h	Increased Gly I and Gly II activities Increased GSH and GSSG contents but decreased GSH/GSSG ratio, increased H_2_O_2_ content and lipid peroxidation	0.5 mM SNP	Increased Gly I activity Increased GSH level as well as the GSH/GSSG ratio Decreased H_2_O_2_ content and lipid peroxidation	[13]
*V. radiata*	6 °C, 2 and 3 days	Increased MG content, H_2_O_2_ content and lipid peroxidation Decreased Gly II activity	0.25 mM Spd	Reduced oxidative stress induced by both MG and ROS decreased GSSG and increased GSH content and GSH/GSSG ratio	[17]
*O. sativa*	4 °C, 8 h	Overexpression of *OsDJ-1C*, increase in Gly III activity Decrease in MG content, increased formation of d-lactate	-	-	[25]
*O. sativa*	4 °C, 15 min–2 h	Overexpression of *OsglyII* and higher accumulation of Gly II	-	-	[99]

**Table 5:** Genetic modifications of glyoxalase genes and their role in conferring abiotic stress tolerance.

Transgenic Plant	Gene	Gene Sources	Tolerance Response in Transgenic Plant	References
*Beta corolliflora × B. vulgaris*	*BvM14-glyoxalase I*	*B. corolliflora* and *B. vulgaris*	Improved tolerance to MG, salt, mannitol and H_2_O_2_ stresses Improved chl content and growth, compared to control under the abiotic stress conditions	[107]
*N. tabacum*	*TaGly I*	*T. aestivum*	Both Gly I and Gly II were overexpressed, which enhanced zinc tolerance Improved chl content, reduced yellowing, and improved phenotype	[105]
*N. tabacum*	*TcGLX1*	*B. juncea*	Increased levels of Gly I, decreased metal accumulation and improved root growth under zinc stress Transgenics also showed similar tolerance response to Cd and Pb	[104]
*N. tabacum*	*Gly I cDNA*	*B. juncea*	Activity of Gly I increased in transgenic plants showed higher tolerance to MG and high salt	[66]
*O. sativa*	*OsDJ-1C*	*Arabidopsis* sp.	Increased Gly III activity, decreased MG content under desiccation stress	[25]
*O. sativa*	*OsDJ-1C*	*A.* *thaliana*	Overexpression of *OsDJ-1C*, increase in Gly III activity, decrease in MG content Increased formation of d-lactate	[25]
*C. Citrange*	*BjGlyI* and *PgGlyII*	*BjGlyI* from *B. juncea* and *PgGlyII* from *Pennisetum glaucum*	Reduced plant yellowing and leaf burn symptom Increased root, shoot and plant dry weight, and reduced accumulation of Na^+^ and Cl^−^ ion, compared to wild type	[106]
*B. juncea*	*Bj glyII*	*P. glaucum*, *O. sativa*, *Arabidopsis*, and *C. arietinum*	Salinity, HM stress, and ABA upregulated the activity of Gly II enzyme	[108]
*O. sativa*	*OsglyII*	*O. sativa*	Activity of Gly II increased under toxic concentrations of MG and NaCl stresses Transgenic plants showed improved growth, increased shoot and root K^+^ content and better ion balance (ratio of Na^+^/K^+^) under salt stress	[67]
*N. tabaccum*	*gly I* and *gly II*	*gly I* from *B. juncea*, *gly II* gene isolated from *O. sativa*	Improved salinity tolerance	[68]
*N. tabacum*	*gly I* and *gly II*	*gly I* from *B. juncea*, *gly II* gene isolated from *O. sativa*	Transgenic plants showed 15% to 50% increase in Gly I activity, and 300% to 400% increase in Gly II activity Reduced toxic Zn accumulation due to maintenance of phytochelatin and GSH content Improved growth, flowering behavior and set normal viable seeds, better yield	[88]
*N. tabacum*	*OsGLYI-11.2*	*O. sativa*	Reduced MG, decreased Na^+^/K^+^ ratio and maintenance of reduced glutathione levels under 200 mM NaCl or 1 mM MG or 5 mM H_2_O_2_	[110]

## Abbreviations

AKR	aldo-keto reductase
ALR	aldose/aldehyde reductase
APX	ascorbate peroxidase
AsA	ascorbate
chl	chlorophyll
CAT	catalase
DHA	dehydroascorbic acid
DHAP	dihydroxyacetone phosphate
DHAR	dehydroascorbate reductase
ETC	electron transport chain
GAP	glyceraldehyde-3-phosphate
Gly	glyoxalase
GR	glutathione reductase
GSH	reduced glutathione
GSSG	oxidized glutathione
GPX	glutathione peroxidase
GST	glutathione *s*-transferase
HT	high temperature
LOX	lipoxygenase
LT	low temperature
MDHAR	monodehydroascorbate reductase
MG	methylglyoxal
MGdG	3-(2′-deoxyribosyl)-6,7-dihydro-6,7-dihydroxy-6/7-methylimidazo-[2,3-*b*]purine-9(8)one
MV	methyl viologen
NADPH	nicotinamide adenine dinucleotide phosphate
PEG	polyethylene glycol
PS II	photosystem II
Pro	proline
ROS	reactive oxygen species
RWC	relative water content
SLG	*s*-d-lactoyl-glutathione
SOD	superoxide dismutase
SNP	sodium nitroprusside
Spd	spermidine
Spm	spermine
Tre	trehalose
